# The Relationship between Changes in *MYBPC3* Single-Nucleotide Polymorphism-Associated Metabolites and Elite Athletes’ Adaptive Cardiac Function

**DOI:** 10.3390/jcdd10090400

**Published:** 2023-09-18

**Authors:** Emna Riguene, Maria Theodoridou, Laila Barrak, Mohamed A. Elrayess, Michail Nomikos

**Affiliations:** 1 College of Medicine, QU Health, Qatar University, Doha P.O. Box 2713, Qatar; emna.riguene@qu.edu.qa (E.R.); lb1802309@student.qu.edu.qa (L.B.); m.elrayess@qu.edu.qa (M.A.E.); 2 Biomedical Research Center (BRC), Qatar University, Doha P.O. Box 2713, Qatar; ma-theodo@hotmail.com

**Keywords:** myosin-binding protein C, heart, endurance, metabolites, elite athlete

## Abstract

Athletic performance is a multifactorial trait influenced by a complex interaction of environmental and genetic factors. Over the last decades, understanding and improving elite athletes’ endurance and performance has become a real challenge for scientists. Significant tools include but are not limited to the development of molecular methods for talent identification, personalized exercise training, dietary requirements, prevention of exercise-related diseases, as well as the recognition of the structure and function of the genome in elite athletes. Investigating the genetic markers and phenotypes has become critical for elite endurance surveillance. The identification of genetic variants contributing to a predisposition for excellence in certain types of athletic activities has been difficult despite the relatively high genetic inheritance of athlete status. Metabolomics can potentially represent a useful approach for gaining a thorough understanding of various physiological states and for clarifying disorders caused by strength–endurance physical exercise. Based on a previous GWAS study, this manuscript aims to discuss the association of specific single-nucleotide polymorphisms (SNPs) located in the *MYBPC3* gene encoding for cardiac MyBP-C protein with endurance athlete status. *MYBPC3* is linked to elite athlete heart remodeling during or after exercise, but it could also be linked to the phenotype of cardiac hypertrophy (HCM). To make the distinction between both phenotypes, specific metabolites that are influenced by variants in the *MYBPC3* gene are analyzed in relation to elite athletic performance and HCM. These include theophylline, ursodeoxycholate, quinate, and decanoyl-carnitine. According to the analysis of effect size, theophylline, quinate, and decanoyl carnitine increase with endurance while decreasing with cardiovascular disease, whereas ursodeoxycholate increases with cardiovascular disease. In conclusion, and based on our metabolomics data, the specific effects on athletic performance for each *MYBPC3* SNP-associated metabolite are discussed.

## 1. Introduction

A talented athlete can be shaped into a champion by a complex interaction of innumerable factors. Understanding elite human performance necessitates the identification of two sources of variation: genes and the environment. As a result, elite athlete performance is a multifactorial trait influenced by genetic predisposition, as well as environmental factors such as nutrition and intense training [1]. Indeed, the development and progression of an elite athlete’s performance are inextricably linked to environmental factors (nutrition and training) as well as genetic predisposition, which determines one’s susceptibility to elite athletic performance (endurance, flexibility, speed, strength, and coordination trainability) and/or multifactorial diseases (diabetes and cancer) [2,3]. Exorbitant training may alter athletes’ blood metabolic profiles. The physical status could be assessed by biochemical, hormonal, and immunological markers. The metabolic demands of athletes depend on the type and intensity of their sports disciplines. Indeed, the duration of training could disturb their cardiovascular function over time and cause some metabolic alterations, such as changes in lipid, glucose, amino acid, and energy metabolites and an increase in plasma lactate and adenine breakdown products [4]. Genotyping enables the evaluation of candidate gene variants that may be associated with elite athlete status. Based on case–control studies, it is possible to determine whether one allele of a DNA sequence (gene or non-coding DNA region) is more common in a group of elite athletes than in the general population and, thus, whether this allele improves performance. The identification of endurance and power markers is based on comparing allelic frequencies between endurance and power athletes, as these two parameters represent opposite extremes of the muscle performance spectrum [5].

DNA polymorphisms are genetic markers that could be associated with a range of phenotypic traits, including endurance, strength, and power of elite athletes. They could account for the interindividual variability in physical performance characteristics in response to endurance or strength training, or other stimuli. Single-nucleotide polymorphisms (SNPs), indels, and structural variations are all examples of DNA variants. Several factors, including the type of polymorphism, its prevalence in a given community, and the total number of athletes studied, are used to assess the importance of a particular sport-related genetic marker [6]. SNPs are used to identify chromosomal regions that may contain genes and sequence variants linked to a variety of traits. Previous research has found that certain loci near a leading SNP have genome-wide significant associations with coronary artery disease, body mass index, C-reactive protein, blood pressure, lipids, and type 2 diabetes mellitus. Some SNPs are linked to the risk of coronary artery disease (CAD), such as genetic variants of the KCNQ1 gene (rs2237892, rs2237895, rs2237897, rs2283228), encoding the potassium voltage-gated channel subfamily Q [7], as well as other CVD phenotypes such as glycemia, inflammation, lipids, and blood pressure [8]. A recent study used the genome-wide association study (GWAS) to investigate the association of common SNPs with endurance athlete status in a large cohort of elite European athletes [1]. By the end of 2020, there were 220 DNA polymorphisms linked to athlete status, with 97 of them found to be significant in at least two studies (35 endurance-related, 24 power-related, and 38 strength-related). However, the underlying molecular mechanisms are poorly characterized [7].

Metabolomics, a relatively new “omics” discipline in addition to “genomics” and “proteomics” in the postgenomic era, could be a good approach to use for a deep understanding of several physiological states and clarification of disorders caused by strength–endurance physical exercise. Metabolomics provides an intermediate phenotype that can better grant a larger effect size, revealing genetic predisposition. Metabolomics is a system biology-based approach to understanding and detecting metabolic changes affected by dietary, lifestyle, and environmental factors. Accordingly, “sportomics” has recently been defined as a new term due to metabolomics’ ability to characterize multiple metabolites in different biological samples simultaneously. This promising tool investigates exercise physiology and exercise-associated metabolism in elite athletes by quantitatively measuring metabolic profiles associated with training [5]. This method enables the identification of biomarkers associated with athlete performance, fatigue response, and potential sports-related disorders [4,6]. This method also enables the detection of differences in metabolic profiles among elite endurance athletes through the identification of serum metabolites involved in fatty acid metabolism, steroid biosynthesis, oxidative stress, and energy-related molecular metabolites. It provides a deep insight into physiological states and clarifies the disorders induced by strength–endurance physical exercise. As a result, the combination of genomics and metabolomics technologies enables more comprehensive coverage of the metabolic pathways involved in complex physiological and pathological processes. For this reason, some studies focusing on the genotyping of selected SNPs in elite athletes participating in various sports disciplines were followed by serum metabolomics to confirm endurance metabolites. The GWAS for metabolic traits (mGWAS) analysis identifies potential SNP-metabolite associations by comparing mGWAS hits in elite athletes to non-elite athletes. The genotyping of 341,385 SNPs in 796 European elite athletes revealed several variants associated with endurance athlete status, but none were significant at the GWAS level. Previous studies demonstrated that rs1052373 is one of the most promising genetic markers [2]. This SNP is found in the myosin-binding protein C3 gene (*MYBPC3*). *MYBPC3* encodes a myosin-associated protein (MyBP-C) found in the cross-bridge bearing zone (C region) of striated muscle A bands. MyBP-C protein phosphorylation influences cardiac contraction. *MYBPC3* mutations have previously been linked to a less super-relaxed state in patients with hypertrophic cardiomyopathy (HCM). Intense exercise can cause heart remodeling to compensate for blood pressure or volume increases by increasing muscle mass. As a result, depending on the type of sport engaged in, the hearts of endurance athletes frequently exhibit eccentric cardiac hypertrophy with increased cavity dimension and wall thickness. Consequently, the endurance-trained heart can deliver a large maximal systolic volume to produce a large cardiac output [2].

In this review, we discuss the potential use of metabolites as valuable tools to better understand the underlying complex molecular pathways that might govern the association of *MYBPC3* SNPs with endurance by leveraging previously published data.

## 2. MyBP-C Structure and Function

**Figure 1:**
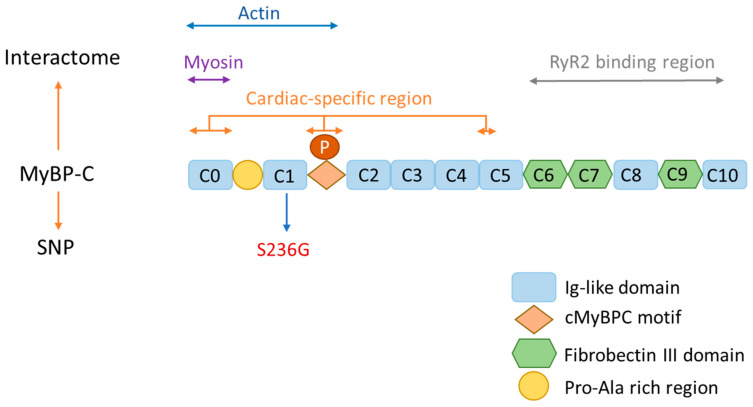
Schematic illustration of cMyBP-C structure and interaction with other sarcomere proteins. The cMyBP-C consists of eight globular Ig-like domains and three Fn-like domains. The N-terminus of cMyBP-C interacts with actin (C0-M) and the myosin-S2 domain (C1-M-C2). C0 and a region of C5 are considered cardiac-specific regions, with the presence of a 28 AA loop. The C-terminus of cMyBP-C interacts with the RyR2 binding region (C6–C10), titin (C8–C10), A-band incorporation (C7–C10), and light meromyosin (LMM) at the C10 domain.

Cardiac muscle contraction occurs when the electrical depolarization of the plasma membrane of the cardiomyocytes causes mechanical contraction of the heart. Actin–myosin cross-bridge cycling regulates the physiological regulation of this contraction. It begins with calcium (Ca^2+^) binding to troponin, which causes tropomyosin to change conformation, allowing myosin head (thick filament) to connect to actin (thin filament). Cardiac MyBP-C (cMyBP-C) is a sarcomere accessory protein, well known as a modulator of cardiomyocyte sarcomere activity [7,9]. It is a multi-domain flexible polypeptide with a molecular mass of ~140 kDa, restricted to the cross-bridge incorporating C zones of striated muscle sarcomeres [10]. Three isoforms exist: slow skeletal (MyBP-C1), fast skeletal (MyBP-C2), and cardiac paralog cMyBP-C, belonging to the immunoglobulin superfamily of intracellular muscle proteins [11,12]. The three isoforms are essential for the regulation of actin–myosin interaction in striated muscles of both the skeletal and heart [11]. Slow and fast paralogs comprise three fibronectin-like domains (Fn) and seven immunoglobulin-like domains (Ig), termed C1 to C10, starting from the N-terminal. For the cardiac isoform, there is an extra domain at the extreme N-terminal termed C0 [10,13], a proline-alanine-rich region between C0 and C1, a phosphorylation motif between C1 and C2, and a 28-amino-acid loop within the C5 domain [9,11]. The N-terminal interacts with actin, myosin, and myosin-S2 (where the myosin head joins the thick filament backbone) [14], and the C-terminal interacts with titin [15], myosin-LMM, A-band incorporation, and the light meromyosin region of the myosin rod [16,17] (Figure 1). Interestingly, in a recent study, it was reported that cardiac ryanodine receptor type 2 (RyR2) in the sarcoplasmic reticulum can directly interact with cMyBP-C, leading to the formation of a cMyBP-C-RyR2 complex that can potentially contribute to the modulation of Ca^2+^ homeostasis in cardiomyocytes [18].

When examined in genetic contexts, the significance of MyBP-C’s role in muscle function becomes even clearer. Genetic variants in sMyBPC1 and fMyBPC2 have been found to induce contractile dysfunction and have been associated with arthrogryposis [19,20].

Similarly, variations in cardiac MyBP-C have been linked to diseases, including inherited hypertrophic cardiomyopathy and dilated cardiomyopathy [13,21]. This emphasizes that MyBP-C impact extends beyond its function and includes factors that affect muscular and cardiac health. Moreover, since MyBP-C plays a role in coordinating muscle contractions, it is involved in regulating the interaction between myosin and actin through the control of the myosin anchoring to the thin filament. Additionally, MyBP-C promotes alignment and synchronization of actin and myosin during muscle contractions through phosphorylation and calcium signaling [22]. This multifaceted functionality highlights its contribution to the mechanical complexities involved in muscle performance.

## 3. MyBP-C and Its Association with Cardiac Disease

Hypertrophic cardiomyopathy (HCM) is a common genetic cardiovascular disorder inherited in an autosomal dominant pattern with varying phenotypes and is often misunderstood in clinical practice. Around 20 million people worldwide are diagnosed with HCM. HCM is distinguished by its variable phenotypic expression, natural history, and genetic profile. With an incidence of 1:500, HCM is the most frequent monogenic cardiovascular disease [23,24].

Indeed, this disease is a genetic heart condition characterized by thickening of the heart muscle. HCM is characterized by an asymmetric decrease in left ventricular (LV) volume, an increase in ejection fraction leading to cardiac myocyte hypertrophy, and disorganization leading to the presence of fibrosis areas [23,25]. The variability of phenotyping is linked to the fact that causal mutations act with other genetic and non-genetic factors. However, 60% of HCM patients are identifiable as having familial disease [26]. 

The identification by Pave et al. of a mutation in MYH7 encoding the sarcomere protein beta-myosin heavy chain opened the door for subsequent findings to search through all sarcomere-encoding genes for causal gene mutations that led to the classification of HCM as a heterogeneous genetic disease [27]. Indeed, mutations in MYH7, cMyBP-C, and other sarcomere proteins like TNNT2, TPM1, MYL2, and MYL3 are the leading causes of HCM [28,29]. Mutations in cMYBP-C can disrupt the normal muscle contraction regulation by affecting its interactions with myosin and actin, crucial for proper contraction [30,31]. These changes lead to impaired relaxation and increased heart muscle stiffness, contributing to the development of HCM [24].

About half of the MYBPC3 mutations lead to shorter protein products, while other mutations cause variations like insertions, deletions, frameshifts, or single amino-acid changes [32]. In HCM patients, most MYBPC3 mutations with premature termination codons reduce protein function, such as frameshift, nonsense, or splice site changes. This suggests that these mutations cause HCM by reducing function through a process called nonsense-mediated RNA decay (NMD) [33]. In NMD, most of the faulty transcript is broken down, leading to lower MyBP-C levels in the sarcomere, which contributes to the condition [34].

HCM caused by MYBPC3 mutations can have variable clinical presentations, ranging from mild to severe. The severity of symptoms and disease progression can vary among individuals with MYBPC3 mutations [32]. It is important to note that HCM is a complex condition influenced by various genetic and environmental factors. While MYBPC3 mutations are a significant contributor to HCM, there are other genes and factors involved in the development and progression of the disease [32].

However, not all genetic variants are causative, as the causality of these variants is linked to the presence of co-segregation and linkage analysis [35]. Missense and loss of function mutations are considered rare causative variants in the general population. These kinds of variants have low phenotypic effects as they depend on other factors, such as environmental and genetic, leading to challenges while studying the causal role of these variants [30,31]. The limitation of genetic testing caused by the rarity of HCM variants and the infrequency of genetic variants makes it difficult to conduct comprehensive segregation studies, leading to many variants being classified as variants of uncertain significance (VUS), where several efforts should be conducted to assess the pathogenicity of these variants through population genetics and functional approaches [2,36].

Understanding the overall effects of MYBPC3 mutations could help us grasp potential outcomes linked to MYBPC mutation. Notably, the mutation in the MYBPC3 gene, located in the exon 30 and known as the rs1052373, has been identified as a common cause of hypertrophic cardiomyopathy (HCM). This mutation can cause HCM by disrupting the normal regulation of muscle contraction [22]. However, its specific function and association with HCM may require further research and investigation.

## 4. MyBP-C and Its Association with Elite Athlete Performance

The association between the *MYBPC3* gene and endurance athlete status has been recently identified. A GWAS study demonstrated an association between *MYBPC3* and increased maximum oxygen uptake (VO_2max_) in elite athlete endurance, suggesting that this protein could play a crucial role in aerobic capacity and endurance performance [2]. To have a clearer insight into the possible influence of the SNP on endurance, it is essential to comprehend how physical activity can affect the cardiovascular system.

Indeed, every type of exercise necessitates an increase in skeletal muscle work. The intensity of exercise (external work) and the body’s demand for oxygen have a direct relationship. Increased pulmonary oxygen uptake (VO_2max_) adheres to the oxygen demand during exercise. Furthermore, the cardiovascular system oversees the transporting of oxygen-rich blood from the lungs to the skeletal muscles, a process measured in liters per minute as cardiac output. The ability of the cardiovascular system to handle the demands of exercising skeletal muscle is improved by exercise-induced remodeling of the heart (EICR). Some structural cardiac changes, such as left ventricular hypertrophy, atrial dilatation, and sport-specific geometry (eccentric vs. concentric), are observed and have been proved by chest radiography, echocardiography, and electrocardiography (ECG) work [37]. These techniques demonstrated that long-term athletic training increases the left ventricular mass. Some changes in cardiac morphology are minor. The effects of training on the heart vary depending on the sport. 

Long-term athletic training can lead to changes in left ventricular wall thickness, cavity dimension, or both, which may be more noticeable in certain sports such as distance running, swimming, cycling, and rowing/canoeing. Athletes training in such sports are more likely to have cardiac disease on their differential diagnosis (Table 1).

The most reliable independent criteria for distinguishing physiologic from pathologic LV hypertrophy was LV cavity size; specifically, LV cavity diameters of 55 mm demonstrated the highest sensitivity and specificity for differentiating an athlete’s heart from HCM. On the other hand, patients with HCM had LV cavity sizes that were within normal limits and never exceeded 55 mm. Absolute LV cavity size increased in athletes with LV hypertrophy, including people who engaged in endurance (rowing, canoeing, and cycling) or combined (water polo and basketball) sports, but not in athletes participating in power disciplines (wrestling and hammer throwing). Furthermore, in the comparison of athletes’ hearts to patients with HCM, the LA size was consistently larger in athletes. This is the fact that atrial enlargement is part of the global cardiac remodeling caused by intense chronic exercise, but it is not associated with the occurrence of supraventricular tachyarrhythmias or poor cardiovascular outcomes. In addition, left atrial enlargement is a common finding in HCM patients and is regarded as a reliable criterion for HCM diagnosis [38].

Vigorous physical activity induces significant adaptative changes in myocardial structure and function, distinguishing it from pathologic hypertrophy. Such discoveries continue to intrigue scientists and clinicians. However, athletes are not immune to cardiovascular symptoms and disease, even though exercise promotes good health. Asymptomatic people may have abnormal values for one of the heart structural or function parameters discussed above. During training, athletes may experience symptoms suggestive of cardiovascular disease [39]. 

It is critical to determine whether the increased left ventricular wall thickness in highly trained athletes is the result of the heart’s physiological adaptation to athletic training or a pathological condition such as HCM [40]. Indeed, athletes with the athlete’s heart typically do not experience symptoms, whereas individuals with HCM may experience symptoms such as chest pain, shortness of breath, or fainting [41]. 

However, when the HCM disease was demonstrated in the athlete’s family history, the distinction between HCM and the athlete’s heart cannot be well determined [40]. 

The most difficult clinical distinction between an athlete’s heart and structural heart disease arises most frequently about HCM because many of the other cardiac disorders that cause sudden death in a young athletic population can be identified independently of any changes in cardiac morphology typically associated with exercise and training [42].

When compared to heart disorders, studying genetics can help us better understand and monitor an athlete’s heart health. Since genetics have such a large influence on how an athlete’s body works, it is critical to figure out how their genes affect their endurance. This allows us to ensure their safety and performance.

**Table 1:** Criteria for recognition between hypertrophy cardiomyopathy (HCM) and the athlete’s heart. Clinical criteria for differentiating physiologic LV hypertrophy from HCM in young athletes [40].

	Elite Athlete’s Endurance	Hypertrophy Cardiomyopathy
Left ventricular thickness	Mild wall thickness	Unusual hypertrophy pattern
Left ventricular cavity size	>55 mm	<45 mm
Left atrium size	≥40 mm	<40 mm
Left ventricular filling	Normal	Abnormal
Family History of HCM	Negative	Positive

## 5. *MYBPC3* SNPs and Endurance

The GWAS is a method based on microarray (SNP-array) analysis that allows for a quick scan of hundreds of thousands of markers across entire sets of DNA from different individuals. This method resulted in the identification of DNA variants that may be associated with a specific trait. GWAS have recently identified various genetic regions showing significant associations with variation in human physical performance traits. These genetic association studies are generally used to assess the impact of variation at a candidate locus on specific performance traits. However, genetic susceptibility studies remain a questionable subject due to the underpowered studies and the small effect size of the identified genetic variants. 

The first report related to GWAS examined the association between many SNPs and the relative rate of maximal oxygen consumption [43]. The validation of the potential functionality of the identified GWAS SNPs was performed through an association study of two SNPs (rs1052373 G and rs7120118 T alleles) with VO_2max_. A previous study conducted a large GWAS study of elite European athletes (753 athletes) and highlighted several novel SNPs linked to endurance. The replication of the top identified SNP associations was carried out in two independent cohorts of elite Russian high and low/moderate aerobic athletes (242 athletes) and elite Japanese endurance athletes (60 athletes), as well as their comparison to combined results. This study found a highly significant link between rs1052373 (*MYBPC3*) and rs7120118 (NR1H3) and endurance in Russian and Japanese people (*p* < 0.05) (Table 2) [2]. The rs1052373 GG (*p* = 1.43 × 10^−8^, odds ratio 2.2) and rs7120118 TT (1.66 × 10^−7^, odds ratio 2) genotypes were found to be overexpressed in high endurance sports at the genome-wide level, as shown in the table below, in addition to the Bonferroni levels of significance. For the combined analysis, the direction of association was similar in all three cohorts, with a similar odds ratio higher than two-fold for all studies except the Russian cohort study [2].

According to a prior GWAS study, the rs1052373 located within the *MYBPC3* gene was significantly associated with enhanced endurance performance among elite athletes in different athlete cohorts (*p* = 1.43 × 10^−8^, odds ratio 2.2) [2]. This suggests that individuals who possess a specific variant of this SNP may have an increased likelihood of being endurance athletes. Certainly, a previous investigation discovered a correlation between this specific SNP and heightened aerobic capacity among endurance athletes. This implies that rs1052373 could potentially impact the body’s capacity to efficiently transport and use oxygen during physical activity—a pivotal element in determining endurance performance [44].

At the protein level, this SNP results in an amino acid change at the 236 position, where a serine (S) has been substituted by a glycine (G) residue (S236G). ClinVar indicates a benign phenotype for this variant. Whether this SNP is causing a mild and not pathological hypertrophy that provided athletes with added advantage needs further investigation to explore the molecular mechanism, if this is the case.

While the exact mechanism is unclear, it is possible that *MYBPC3* gene polymorphisms, including SNP rs1052373, may affect cardiac function and remodeling in elite athletes. Accordingly, further research is needed to fully understand the relationship between *MYBPC3* gene polymorphisms and cardiac function and remodeling in elite athletes.

Indeed, prior research has indicated that athletes’ performance may be associated with changes in their metabolic profile, depending on the physical activity [37]. An in-depth examination through a GWAS study unveiled a multitude of SNPs related to higher endurance, and these were found to be associated with specific metabolites [2]. 

Another study discovered that the genetic variation rs1052373 was associated with higher levels of the testosterone precursor androstenediol disulfate. It implies that such genetic variation could affect the process of steroid metabolism, potentially influencing both muscle development and endurance performance. Consequently, endurance athletes often have higher aerobic capacity and exhibit a different steroid metabolism than non-athletes [2].

As the relationship between sports activity and an athlete’s cardiac physiology and function remains unclear, we hypothesize that the SNP may have a secondary effect on cardiac function through some metabolites, which are produced under the control of *MYBPC3* and related SNP. 

Prior research demonstrated that the hearts of endurance athletes show some adaptations to intense training [37]. These adaptations, however, may be linked to an increased risk of cardiovascular disease. Indeed, a recent study found that endurance athletes with high cardiovascular demands (higher blood pressure and stroke volume) have a metabolic signature that indicates a higher risk of cardiovascular disease.

## 6. *MYBPC3* SNP-Associated Metabolites

During or after exercise or training, the athlete’s body produces small molecules called metabolites through metabolic processes. The metabolomics approach can offer an investigation of metabolites potentially associated with adaptive heart remodeling due to exercise in comparison to patients with cardiovascular diseases (CVD), including HCM. The analysis of metabolites present in various biological samples, such as urine, blood, and saliva, helps scientists gain insights into the metabolic changes occurring in response to exercise and physical activity (Table 3) [45]. 

Prior research confirms that exercise triggers an immediate change in metabolism, affecting pathways linked to cardiometabolic health and cardiovascular disease. Significantly, alterations in amino acid and lipid metabolism were observed following subacute endurance exercise, while modifications in glycolysis, TCA cycle and nucleotide metabolism occurred following chronic endurance exercise. The reported metabolic pathways are closely connected. They have interconnected roles in aerobic and anaerobic respiration, fatty acid oxidation, branched-chain amino acid catabolism, and oxidative stress [45]. 

In a previous study, notable metabolic pathways, such as the glutamate pathway, showed a significant increase, suggesting reduced cardiometabolic risk. Additionally, changes in the urea cycle were observed due to exercise-induced inflammation.

Furthermore, the study revealed a general reduction in metabolite levels connected to insulin resistance and cardiovascular risk, accompanied by an increase in those associated with elevated inflammation [45].

For further understanding of the association of rs1052373 with athletes ‘endurance, several metabolites were studied to assess their association with the selected SNP. Four metabolites were suggested to be associated with *MYBPC3* expression and potentially linked to elite athletes’ endurance: quinate, theophylline, decanoyl-carnitine, and ursodeoxycholic acid [37]. Although there may be no direct link between *MYBPC3* expression and these metabolites, SNPs within *MYBPC3* can indirectly shape metabolites through pleiotropy, pathway analysis, linkage disequilibrium proxy, and spatial associations [52]. Elite athletes’ intensive physical training may cause physiological adaptation in the cardiovascular system, resulting in increased stroke volume and blood pressure to optimize performance. While exercise has been linked to improved health outcomes, it can also increase the risk of heart-related conditions like arrhythmias, myocardial infarction, aortic dissection, and sudden cardiac arrest [4,53]. According to Table 4, theophylline, quinate, and decanoyl carnitine increase with endurance while decreasing with cardiovascular disease, but ursodeoxycholate increases with CVD [4,54]. 

Understanding the role of these four metabolites in the athlete’s cardiac function led to a better understanding of their role in the endurance and performance of elite athletes. Each metabolite’s function is discussed further below.

### 6.1. Quinate

Quinate is produced by many plants, including fruits, vegetables, and herbal remedies, with coffee and maté being particularly rich dietary sources. Previous research demonstrated that this metabolite could inhibit inflammatory activation and oxidative stress in macrophages [17]. The majority of the quinate derivatives inhibited the 2,2-diphenyl-1-picrylhydrazyl (DPPH) radical scavenging, superoxide anion scavenging, and lipid peroxidation. It also significantly inhibited lipid peroxidation [18], and it had significant inhibitory activity against the hepatitis B virus DNA (HBV-DNA) replication in HepG2.2.15 cells [19]. Quinate is also a pharmacodynamic marker involved in the prevention of heart failure, in addition to its antiviral effect in vitro [16]. The antioxidant properties may protect against many diseases, such as diabetes, cardiovascular diseases, inflammation, pulmonary diseases, or aging [38]. This metabolite is used to treat muscle cramps that occur during exercise in athletes. High levels of quinine were found in soccer players. It inhibits nerve stimulation by decreasing the excitability of the motor end plate and increasing the refractory period of skeletal muscular contraction [59,60].

### 6.2. Theophylline

Theophylline occurs naturally in tea and cocoa beans in trace amounts. It is metabolized by the cytochrome P450 enzymes. It is used as a treatment for asthma and chronic obstructive pulmonary disease (COPD) (positive cardiovascular actions). Theophylline is known to relax the smooth muscles in the bronchial airways and pulmonary blood vessels. It reduces pulmonary artery pressures and pulmonary vascular resistance, in addition to increasing both right and left ventricular ejection fractions [59]. This metabolite exerts these effects through different mechanisms. Firstly, theophylline acts as a competitive nonselective phosphodiesterase inhibitor (inhibiting type III and type IV phosphodiesterase), reducing inflammation [60]. It does increase the release of interleukin 10 (IL-10), which is known for its broad spectrum of anti-inflammatory effects by inhibiting phosphodiesterase (PDE) [61]. This latter resulted in the inhibition of cyclic AMP enzymatic degradation by PDE, which increased the heart rate as well as the force of contraction and rate of relaxation. These latter are critical components of the response required for the heart to increase its stroke volume when the heart rate is increased. Secondly, theophylline also acts as a nonselective adenosine receptor antagonist, acting on A1, A2, and A3 receptors with almost the same affinity, which potentially explains its cardiac effects. Adenosine-mediated channels also increase the contraction force of diaphragmatic muscles by enhancing their Ca^2+^ uptake [9]. In addition, theophylline, like caffeine, stimulates diuresis and may have some metabolic effects, the most notable of which is increased lipolysis via increased lipid mobilization from adipose tissues, resulting in increased carnitine transport into renal tissues to form palmitoyl-carnitine groups for subsequent oxidation inside the mitochondria [6]. It may also have an antioxidant effect by inhibiting oxidative stress, which is seen in the bronchopulmonary inflammation process in asthmatics [5]. Some of theophylline’s pharmacodynamic actions are similar to those of caffeine. It was discovered to increase endurance in the same way that caffeine does, explaining its high levels in high-power athletes. Greer et al. demonstrated that theophylline increased endurance time in a 32 min exercise [62]. This metabolite, on the other hand, had no direct effect on VO_2max_, muscle parameters, or ventilatory parameters. In terms of benefits, theophylline can be used as a supplement in its pure form but only with prescription.

### 6.3. Decanoyl-Carnitine 

Decanoyl-carnitine belongs to the acyl-carnitines class of compounds. It is, more specifically, a decanoic acid ester of carnitine. It is assumed that the human body contains over 1000 different types of acyl-carnitines. Decanoyl carnitine was a major plasma metabolite that distinguished healthy and diabetic men. It is positively correlated with oxidized low-density lipoprotein cholesterol (LDL), 8-epi-PGF2α, IL-6, and the tumor necrosis factor (TNF-α) [10]. This metabolite is significantly higher in stroke patients with a high risk of cardio-embolism than in those with a low or intermediate risk (*p* = 0.040). It is associated with high-sensitivity C-reactive protein (CRP) but not with high-density lipoprotein cholesterol (HDL) [63].

This metabolite is a fatty acid-oxidation intermediate and a marker for incomplete fatty acid oxidation [64]. According to functional metabolomics, it is a dominant biomarker during a moderately intense exercise bout capable of promoting fat oxidation. This physiological acylcarnitine production and efflux could have biological benefits in muscle tissue [6]. When the influx of acyl-CoA into mitochondria exceeds the capacity for complete fatty acid oxidation due to chronic substrate oversupply, acylcarnitine efflux occurs. On the other hand, the increased availability of “oxidation” substrates is a physiological phenomenon linked to high lipolytic rates during exercise or starvation, resulting in acute acylcarnitine efflux into plasma as total esterified carnitines or total long-chain acylcarnitine [6].

Indeed, this metabolite improves fatty acid transport into the mitochondria, making exercise more productive. The body is unable to convert medium-chain fatty acids into acetyl-CoA and then to energy via the tricarboxylic acid (TCA) cycle, which is linked to oxidative phosphorylation in the respiratory chain in the case of some metabolic diseases. The presence of specific metabolites in the blood, particularly octanoyl- and decanoyl-carnitine, easily identifies the enzymatic defect. Furthermore, energy deficiency and some metabolic disorders have underlying pathophysiological mechanisms related to the production of reactive oxygen species (ROS), which have an impact on athletes’ physical performance. Indeed, metabolomics analysis shows an increase in fatty acid oxidation products such as acylcarnitine after long-term exercise, as seen in CVD patients. Previous research discovered that higher levels of decanoyl-carnitine may indicate glycogen depletion, which is frequently associated with fatigue, implying that glycogen resynthesis should be a metabolic priority [65,66]. Previous research found that this metabolite was upregulated in elderly and adult subjects (increased with age) [64]. As a result, carnitine supplementation may benefit athletes by improving exercise performance. It raises maximal oxygen consumption while decreasing the respiratory quotient, implying a possible role in lipid metabolism stimulation. After a long training session, this metabolite could significantly lower plasma lactate levels. The addition of such a metabolite can reduce the negative effects of hypoxic training while also accelerating recovery from exercise stress. As a result, there is evidence that L-carnitine supplementation benefits training, competition, and recovery from strenuous exercise, as well as regenerative athletics. This metabolite may be associated with an increased risk of CVD in men, particularly coronary artery disease and heart failure in general [54]. This explains why this metabolite had a high *p*-value in the CVD study compared to the GWAS study.

### 6.4. Ursodeoxycholic Acid

Bacteria in the intestine produce ursodeoxycholic acid (UDCA). It has a hydroxyl group in a cyclic chair conformation, making it more stable than its isomer, chenodeoxycholic acid (CDCA). UDCA is the least toxic bile acid, which is synthesized by dihydroxylation of the free bile acid CDCA [67]. UDCA is known as a cholesterol-lowering agent and anti-inflammatory metabolite. As a bile acid, it is reported to have a vasodilator effect. Thus, it is used for the treatment of patients suffering from coronary heart disease. Indeed, patients with chronic heart failure have altered ratios of primary and secondary bile acids, and elevated bile acids have been linked to arrhythmias in adult and fetal hearts. According to a previous study, UDCA can also protect the heart from taurocholate-induced arrhythmias [68]. UDCA can also maintain normal intracellular [Ca^2+^] dynamics against hypoxic conditions, which are also mediated by Gi-coupled receptors [69,70]. This bile acid also shows a cardioprotective effect, like dexamethasone, through the alteration in the bile acid transporter gene’s expression and metabolism [54]. Bile acid concentrations were found to be significantly lower in athletes compared to sedentary men in the control group. This difference has been explained by the fact that bile acids in plasma were found to be negatively correlated with cognitive restraint of eating, implying that their metabolism undergoes a compensatory adaptation to prevent further overeating after exercise. Finally, exercise can alter cardiovascular function by lowering heart rate and blood pressure while increasing maximal myocardial oxygen uptake. It also has an impact on skeletal and cardiac muscle, blood volume, and several metabolic processes [39]. High-intensity resistance training increases muscular strength, and new research indicates that metabolite accumulation plays a role in this process. 

Certainly, metabolites can have secondary effects on an athlete’s endurance through their influence on various physiological processes. These outcomes can shape an athlete’s performance in several ways. For instance, during training, metabolites actively contribute to energy production, impacting an athlete’s ability to sustain physical effort. 

When accumulated, certain metabolites, such as lactate, could induce muscle fatigue, ultimately curtailing endurance. Furthermore, metabolites exert control over the transition between aerobic and anaerobic metabolism during exercise phases to maintain endurance.

In the realm of hormonal regulation, certain metabolites can impact hormonal regulation, including those related to energy metabolism and stress response, which is crucial to maintaining endurance and good performance for athletes.

However, it is worth noting that some metabolites generated during training have the potential to incite inflammatory responses. Overbearing inflammation can impede recovery and hinder an athlete’s participation in subsequent training sessions.

Physiological adaptation, particularly within muscle, also relies on metabolite production to increase mitochondrial function and improve endurance capacity. Additionally, neuromuscular communication is also under the control of metabolites, underpinning muscle contraction and streamlined movement, thereby contributing to overall endurance performance. Metabolites also help keep a balance that reduces stress and keeps cells working well during exercise.

Given these intricate interactions, understanding how metabolites impact the multifaceted aspects of endurance yields can provide deep insights into an athlete’s performance limitations, recovery requisites, and potential areas for improvement. 

Understanding optimal endurance extends beyond athletics, resonating with broader health and wellness concerns within communities. As such, the goal of this review is to consolidate current knowledge about endurance-related metabolites in athletes alongside the genetic variants (SNPs) influencing their production. Environmental factors such as diet and nutrition should be considered to improve an athlete’s endurance and performance [39].

## 7. Limitations, Future Work, and Concluding Remarks

*MYBPC3* SNP-related metabolites may improve cardiac function by lowering inflammation, oxidative stress, and lipid peroxidation, as well as promoting fatty acid oxidation, energy metabolism, and maintaining normal intracellular calcium dynamics. This, in turn, contributes to better endurance and performance in elite athletes. *MYBPC3* appears to have paradoxical effects on the risk of cardiovascular disease. Depending on the other proteins or genes under its control, each *MYBPC3* SNP may have a different effect on cardiac function.

In any case, even if a specific SNP in the *MYBPC3* gene is shown to be truly beneficial for elite endurance and heart function, there are other variants associated with cardiac pathology. Longitudinal studies to assess changes in the levels of these metabolites over time and their impact on athletic performance are needed to determine whether the relationship between these metabolites, particularly decanoyl-carnitine and athletic performance, is causal or merely correlational. Furthermore, experimental studies are being conducted to investigate the effects of manipulating these metabolites on exercise capacity and other performance measures. We must also acknowledge the limitations, such as the investigation of potential confounding factors such as diet, training status, and genetic factors, which may aid in clarifying the relationship between decanoyl-carnitine and athletic performance and determining whether the observed association is a direct causal effect or an indirect association mediated by other factors. We must also demonstrate how the meta-analysis approach, which included the collection and comparison of metabolomics data, aided in the identification of the specific effects of these metabolites on athletic performance. To summarize, we suggest that *MYBPC3* SNPs and their associated metabolites may improve cardiac function and remodeling in elite athletes. However, further investigation is needed to shed light and delineate this interesting phenomenon.

## Figures and Tables

**Table 2:** SNPs linked to endurance athlete status were discovered, replicated, and meta-analyzed [2].

	rs 1052373		rs 7120118	
Genotype	GG		TT	
GWAS	*p* OR (95% CI)	5.48 × 10^−6^ 2.61 (1.72–3.94)	*p* OR (95% CI)	1.26 × 10^−5^ 2.49 (1.65–3.75)
Russian cohort	*p* OR (95% CI)	1.2 × 10^−2^ 1.67 (1.12–2.49)	*p* OR (95% CI)	1.6 × 10^−2^ 1.64 (1.10–2.45)
Japanese cohort	*p* OR (95% CI)	2.7 × 10^−3^ 2.92 (1.41–6.05)	*p* OR (95% CI)	3.52 × 10^−2^ 2.48 (1.10–5.56)
Combined	*p* OR (95% CI)	1.43 × 10^−8^ 2.17 (1.67–2.84)	*p* OR (95% CI)	1.66 × 10^−7^ 2.07 (1.59–2.7)

**Table 3:** Metabolic changes associated with endurance exercise [45].

Exercise	Findings	Reference
Trans Japan Alps race	Increase in lipid metabolism and hemolysis.	[46]
Endurance exercise (bike)	Significant alterations in metabolites linked to cellular energy processes. Induction of metabolites associated with glycolytic pathways.	[47]
Shuttle runs	Significant upregulation of metabolites associated with amino acids after exercise. Downregulation of steroid hormone metabolism after exercise.	[48]
Endurance and speed endurance training	Exercise leads to significant changes in metabolism. Before and after exercise, there is a rise in lactic acid and glycine levels while the concentration of creatinine decreases.	[49]
Cardiopulmonary exercise	Reduction in produced metabolites associated with insulin resistance. Increase in metabolites linked to lipolysis.	[50]
Outdoor running	Activation of metabolic pathways of amino acid and fatty acid metabolism. Mitochondrial and metabolic changes induced by ATP signaling.	[51]

**Table 4:** List of metabolites potentially associated with the elite athletes’ endurance.

Metabolite	rsID	SNPpos	HapMap Allele	Beta GWAS Server	*p*-Value	Reference
Theophylline	10769255	chr11:47367371	C/T	0.0523	5.217 × 10^−4^	[55]
Urso-deoxycholate	rs10838696	chr11:47363285	G/A	0.04233	8.696 × 10^−4^	[56]
Quinate	rs2856650	chr11:47365199	C/T	−0.082	2.257 × 10^−4^	[57]
Decanoyl-carnitine	rs11570058	chr11:47369443	C/T	−0.0232	6.64 × 10^−5^	[58]

## Abbreviations

DNA—deoxyribonucleic acid; SNP—single-nucleotide polymorphism; CAD—coronary artery disease; KCNQ1—potassium voltage-gated channel subfamily Q member 1; GWAS—genome-wide association study; mGWAS—metabolic genome-wide association study; *MYBPC3*—myosin-binding protein C3 gene; MyBP-C—myosin-binding protein C3; HCM—hypertrophic cardiomyopathy; Fn domain—fibronectin-like domains; Ig domain—immunoglobulin-like domains; RyR2—calcium channel ryanodine receptor 2; LV—left ventricular; MYH7—sarcomere protein beta-myosin heavy chain gene; VUS—variants of uncertain significance; EICR—exercise-induced remodeling of the heart; ECG—electro-cardiography; CVDs—cardiovascular diseases; DPPH—2,2-diphenyl-1-picrylhydrazyl; HBV-DNA—hepatitis B virus DNA; COPD—chronic obstructive pulmonary disease; IL-10—interleukin 10; PDE—phosphodiesterase; LDL—low-density lipoprotein cholesterol; TNF—tumor necrosis factor; CRP—C-reactive protein; HDL—high-density lipoprotein cholesterol; TCA—tricarboxylic acid; ROS—reactive oxygen species; UDCA—ursodeoxycholic acid; CDCA—chenodeoxycholic acid.

## References

[1] Georgiades E., Klissouras V., Baulch J., Wang G., Pitsiladis Y. (2017). Why Nature Prevails over Nurture in the Making of the Elite Athlete. BMC Genom..

[2] Al-Khelaifi F., Yousri N.A., Diboun I., Semenova E.A., Kostryukova E.S., Kulemin N.A., Borisov O.V., Andryushchenko L.B., Larin A.K., Generozov E.V. (2020). Genome-Wide Association Study Reveals a Novel Association between MYBPC3 Gene Polymorphism, Endurance Athlete Status, Aerobic Capacity and Steroid Metabolism. Front. Genet..

[3] MacArthur D.G., North K.N. (2005). Genes and Human Elite Athletic Performance. Hum. Genet..

[4] Al-Khelaifi F., Donati F., Botre F., Latiff A., Abraham D., Hingorani A., Georgakopoulos C., Suhre K., Yousri N.A., Elrayess M.A. (2019). Metabolic Profiling of Elite Athletes with Different Cardiovascular Demand. Scand. J. Med. Sci. Sports.

[5] Znazen H., Mejri A., Touhami I., Chtara M., Siala H., LE Gallais D., Ahmetov I.I., Messaoud T., Chamari K., Soussi N. (2016). Genetic Advantageous Predisposition of Angiotensin Converting Enzyme Id Polymorphism in Tunisian Athletes. J. Sports Med. Phys. Fit..

[6] Ahmetov I.I., Hall E.C.R., Semenova E.A., Pranckevičienė E., Ginevičienė V. (2022). Advances in Sports Genomics. Adv. Clin. Chem..

[7] Bers D.M. (2002). Cardiac Excitation-Contraction Coupling. Nature.

[8] Clark K.A., McElhinny A.S., Beckerle M.C., Gregorio C.C. (2002). Striated Muscle Cytoarchitecture: An Intricate Web of Form and Function. Annu. Rev. Cell Dev. Biol..

[9] Moss R.L., Fitzsimons D.P., Ralphe J.C. (2015). Cardiac MyBP-C Regulates the Rate and Force of Contraction in Mammalian Myocardium. Circ. Res..

[10] Flashman E., Redwood C., Moolman-Smook J., Watkins H. (2004). Cardiac Myosin Binding Protein C: Its Role in Physiology and Disease. Circ. Res..

[11] Lin B., Govindan S., Lee K., Zhao P., Han R., Runte K.E., Craig R., Palmer B.M., Sadayappan S. (2013). Cardiac Myosin Binding Protein-C Plays No Regulatory Role in Skeletal Muscle Structure and Function. PLoS ONE.

[12] Sadayappan S., de Tombe P.P. (2012). Cardiac Myosin Binding Protein-C: Redefining Its Structure and Function. Biophys. Rev..

[13] Barefield D., Sadayappan S. (2010). Phosphorylation and Function of Cardiac Myosin Binding Protein-C in Health and Disease. J. Mol. Cell Cardiol..

[14] Singh R.R., McNamara J.W., Sadayappan S. (2021). Mutations in Myosin S2 Alter Cardiac Myosin-Binding Protein-C Interaction in Hypertrophic Cardiomyopathy in a Phosphorylation-Dependent Manner. J. Biol. Chem..

[15] Zacharchenko T., von Castelmur E., Rigden D.J., Mayans O. (2015). Structural Advances on Titin: Towards an Atomic Understanding of Multi-Domain Functions in Myofilament Mechanics and Scaffolding. Biochem. Soc. Trans..

[16] Okagaki T., Weber F.E., Fischman D.A., Vaughan K.T., Mikawa T., Reinach F.C. (1993). The Major Myosin-Binding Domain of Skeletal Muscle MyBP-C (C Protein) Resides in the COOH-Terminal, Immunoglobulin C2 Motif. J. Cell Biol..

[17] Inchingolo A.V., Previs S.B., Previs M.J., Warshaw D.M., Kad N.M. (2019). Revealing the Mechanism of How Cardiac Myosin-Binding Protein C N-Terminal Fragments Sensitize Thin Filaments for Myosin Binding. Proc. Natl. Acad. Sci. USA.

[18] Stanczyk P.J., Seidel M., White J., Viero C., George C.H., Zissimopoulos S., Lai F.A. (2018). Association of Cardiac Myosin-Binding Protein-C with the Ryanodine Receptor Channel—Putative Retrograde Regulation?. J. Cell Sci..

[19] Bayram Y., Karaca E., Coban Akdemir Z., Yilmaz E.O., Tayfun G.A., Aydin H., Torun D., Bozdogan S.T., Gezdirici A., Isikay S. (2016). Molecular Etiology of Arthrogryposis in Multiple Families of Mostly Turkish Origin. J. Clin. Investig..

[20] Desai D., Stiene D., Song T., Sadayappan S. (2020). Distal Arthrogryposis and Lethal Congenital Contracture Syndrome—An Overview. Front. Physiol..

[21] Kuster D.W., Bawazeer A.C., Zaremba R., Goebel M., Boontje N.M., van der Velden J. (2012). Cardiac Myosin Binding Protein C Phosphorylation in Cardiac Disease. J. Muscle Res. Cell Motil..

[22] Heling LW H.J., Geeves M.A., Kad N.M. (2020). MyBP-C: One Protein to Govern Them All. J. Muscle Res. Cell Motil..

[23] Maron B.J., Rowin E.J., Casey S.A., Maron M.S. (2016). How Hypertrophic Cardiomyopathy Became a Contemporary Treatable Genetic Disease with Low Mortality: Shaped by 50 Years of Clinical Research and Practice. JAMA Cardiol..

[24] Seidman C.E., Seidman J.G. (2011). Identifying Sarcomere Gene Mutations in Hypertrophic Cardiomyopathy: A Personal History. Circ. Res..

[25] Adalsteinsdottir B., Burke M., Maron B.J., Danielsen R., Lopez B., Diez J., Jarolim P., Seidman J., Seidman C.E., Ho C.Y. (2020). Hypertrophic Cardiomyopathy in Myosin-Binding Protein C (MYBPC3) Icelandic Founder Mutation Carriers. Open Heart.

[26] Hartmannova H., Kubanek M., Sramko M., Piherova L., Noskova L., Hodanova K., Stranecky V., Pristoupilova A., Sovova J., Marek T. (2013). Isolated X-Linked Hypertrophic Cardiomyopathy Caused by a Novel Mutation of the Four-and-a-Half LIM Domain 1 Gene. Circ. Cardiovasc. Genet..

[27] Cirino A.L., Harris S., Lakdawala N.K., Michels M., Olivotto I., Day S.M., Abrams D.J., Charron P., Caleshu C., Semsarian C. (2017). Role of Genetic Testing in Inherited Cardiovascular Disease: A Review. JAMA Cardiol..

[28] Maron B.J., Maron M.S., Maron B.A., Loscalzo J. (2019). Moving Beyond the Sarcomere to Explain Heterogeneity in Hypertrophic Cardiomyopathy: JACC Review Topic of the Week. J. Am. Coll. Cardiol..

[29] Maron B.J., Maron M.S., Maurer M.S., Rowin E.J., Maron B.A., Galie N. (2021). Cardiovascular Diseases That Have Emerged From the Darkness. J. Am. Heart Assoc..

[30] Ingles J., Doolan A., Chiu C., Seidman J., Seidman C., Semsarian C. (2005). Compound and Double Mutations in Patients with Hypertrophic Cardiomyopathy: Implications for Genetic Testing and Counselling. J. Med. Genet..

[31] Ingles J., Yeates L., Semsarian C. (2011). The Emerging Role of the Cardiac Genetic Counselor. Heart Rhythm..

[32] Tudurachi B.-S., Zăvoi A., Leonte A., Țăpoi L., Ureche C., Bîrgoan S.G., Chiuariu T., Anghel L., Radu R., Sascău R.A. (2023). An Update on MYBPC3 Gene Mutation in Hypertrophic Cardiomyopathy. Int. J. Mol. Sci..

[33] Carrier L. (2019). Making Sense of Inhibiting Nonsense in Hypertrophic Cardiomyopathy. Circulation.

[34] Helms A.S., Tang V.T., O’Leary T.S., Friedline S., Wauchope M., Arora A., Wasserman A.H., Smith E.D., Lee L.M., Wen X.W. (2020). Effects of MYBPC3 Loss-of-Function Mutations Preceding Hypertrophic Cardiomyopathy. JCI Insight.

[35] Kelly M., Semsarian C. (2009). Multiple Mutations in Genetic Cardiovascular Disease: A Marker of Disease Severity?. Circ. Cardiovasc. Genet..

[36] Ahluwalia M., Ho C.Y. (2021). Cardiovascular Genetics: The Role of Genetic Testing in Diagnosis and Management of Patients with Hypertrophic Cardiomyopathy. Heart.

[37] Al-Khelaifi F., Diboun I., Donati F., Botrè F., Alsayrafi M., Georgakopoulos C., Suhre K., Yousri N.A., Elrayess M.A. (2018). A Pilot Study Comparing the Metabolic Profiles of Elite-Level Athletes from Different Sporting Disciplines. Sports Med.-Open.

[38] Gomez-Cabrera M.C., Domenech E., Vina J. (2008). Moderate Exercise Is an Antioxidant: Upregulation of Antioxidant Genes by Training. Free Radic. Biol. Med..

[39] Baggish A.L., Wood M.J. (2011). Athlete’s Heart and Cardiovascular Care of the Athlete. Circulation.

[40] Maron B.J. (2005). Distinguishing Hypertrophic Cardiomyopathy from Athlete’s Heart: A Clinical Problem of Increasing Magnitude and Significance. Heart.

[41] Prutkin J.M., Ackerman M.J., Drezner J.A. (2016). Athletes with Implantable Cardioverter Defibrillators: Can They Return to Competitive Sports?. Heart.

[42] Campbell R.M., Berger S., Drezner J. (2009). Sudden Cardiac Arrest in Children and Young Athletes: The Importance of a Detailed Personal and Family History in the Pre-Participation Evaluation. Br. J. Sports Med..

[43] Al-Khelaifi F., Diboun I., Donati F., Botrè F., Abraham D., Hingorani A., Albagha O., Georgakopoulos C., Suhre K., Yousri N.A. (2019). Metabolic GWAS of Elite Athletes Reveals Novel Genetically-Influenced Metabolites Associated with Athletic Performance. Sci. Rep..

[44] Verwijs S.M., Pinto Y.M., Kuster D.W., van der Velden J., Limpens J., van Hattum J.C., van der Crabben S.N., Lekanne Deprez R.H., Wilde A.A., Jørstad H.T. (2022). Beneficial Effects of Cardiomyopathy-Associated Genetic Variants on Physical Performance: A Hypothesis-Generating Scoping Review. Cardiology.

[45] Jaguri A., Al Thani A.A., Elrayess M.A. (2023). Exercise Metabolome: Insights for Health and Performance. Metabolites.

[46] Yamagata T., Sakuraba K. (2019). Changes in Urine Components and Characteristics During a 415-Km Mountain Ultra-Marathon. Juntendo Med. J..

[47] Morville T., Sahl R.E., Moritz T., Helge J.W., Clemmensen C. (2020). Plasma Metabolome Profiling of Resistance Exercise and Endurance Exercise in Humans. Cell Rep..

[48] Zhao J., Wang Y., Zhao D., Zhang L., Chen P., Xu X. (2020). Integration of Metabolomics and Proteomics to Reveal the Metabolic Characteristics of High-Intensity Interval Training. Analyst.

[49] Chen Y., Wang X. (2022). Analysis of Metabonomic Characteristics after Exercise Fatigue Based on NMR. Contrast Media Mol. Imaging.

[50] Nayor M., Shah R.V., Miller P.E., Blodgett J.B., Tanguay M., Pico A.R., Murthy V.L., Malhotra R., Houstis N.E., Deik A. (2020). Metabolic Architecture of Acute Exercise Response in Middle-Aged Adults in the Community. Ciculation.

[51] Li K., Schön M., Naviaux J.C., Monk J.M., Alchus-Laiferová N., Wang L., Straka I., Matejička P., Valkovič P., Ukropec J. (2022). Cerebrospinal Fluid and Plasma Metabolomics of Acute Endurance Exercise. FASEB J..

[52] Gallois A., Mefford J., Ko A., Vaysse A., Julienne H., Ala-Korpela M., Laakso M., Zaitlen N., Pajukanta P., Aschard H. (2019). A Comprehensive Study of Metabolite Genetics Reveals Strong Pleiotropy and Heterogeneity across Time and Context. Nat. Commun..

[53] Lawless C.E., Olshansky B., Washington R.L., Baggish A.L., Daniels C.J., Lawrence S.M., Sullivan R.M., Kovacs R.J., Bove A.A. (2014). Sports and exercise cardiology in the United States: Cardiovascular specialists as members of the athlete healthcare team. J. Am. Coll. Cardiol..

[54] Zhao J.V., Burgess S., Fan B., Schooling C.M. (2022). L-Carnitine, a Friend or Foe for Cardiovascular Disease? A Mendelian Randomization Study. BMC Med..

[55] Matthay R.A. (1985). Effects of Theophylline on Cardiovascular Performance in Chronic Obstructive Pulmonary Disease. Chest.

[56] Tousoulis D., Papageorgiou N., Stefanadis C. (2012). Ursodeoxycholic acid in patients with chronic heart failure. J. Am. Coll. Cardiol..

[57] Nickolas T.L. (2023). Treating Osteoporosis with Denosumab in Patients on Hemodialysis: The Good, the Bad, and the Ugly. Clin. J. Am. Soc. Nephrol..

[58] Li Y., Sekula P., Wuttke M., Wahrheit J., Hausknecht B., Schultheiss U.T., Gronwald W., Schlosser P., Tucci S., Ekici A.B. (2018). Genome-Wide Association Studies of Metabolites in Patients with CKD Identify Multiple Loci and Illuminate Tubular Transport Mechanisms. J. Am. Soc. Nephrol..

[59] Kennedy M. (2021). Effects of Theophylline and Theobromine on Exercise Performance and Implications for Competition Sport: A Systematic Review. Drug Test. Anal..

[60] Chorostowska-Wynimko J., Kus J., Skopińska-Rózewska E. (2007). Theophylline Inhibits Free Oxygen Radicals Production by Human Monocytes via Phosphodiesterase Inhibition. J. Physiol. Pharmacol..

[61] McLellan T.M., Caldwell J.A., Lieberman H.R. (2016). A Review of Caffeine’s Effects on Cognitive, Physical and Occupational Performance. Neurosci. Biobehav. Rev..

[62] Greer F., Friars D., Graham T.E. (2000). Comparison of Caffeine and Theophylline Ingestion: Exercise Metabolism and Endurance. J. Appl. Physiol. (1985).

[63] Seo W.-K., Jo G., Shin M.-J., Oh K. (2018). Medium-Chain Acylcarnitines Are Associated with Cardioembolic Stroke and Stroke Recurrence. Arterioscler. Thromb. Vasc. Biol..

[64] Saud Gany S.L., Tan J.K., Chin K.Y., Hakimi N.H., Ab Rani N., Ihsan N., Makpol S. (2022). Untargeted Muscle Tissue Metabolites Profiling in Young, Adult, and Old Rats Supplemented with Tocotrienol-Rich Fraction. Front. Mol. Biosci..

[65] Shulman R.G., Rothman D.L. (2001). The “Glycogen Shunt” in Exercising Muscle: A Role for Glycogen in Muscle Energetics and Fatigue. Proc. Natl. Acad. Sci. USA.

[66] Pla R., Pujos-Guillot E., Durand S., Brandolini-Bunlon M., Centeno D., Pyne D.B., Toussaint J.F., Hellard P. (2021). Non-Targeted Metabolomics Analyses by Mass Spectrometry to Explore Metabolic Stress after Six Training Weeks in High Level Swimmers. J. Sports Sci..

[67] Hanafi N.I., Mohamed A.S., Sheikh Abdul Kadir S.H., Othman M.H.D. (2018). Overview of Bile Acids Signaling and Perspective on the Signal of Ursodeoxycholic Acid, the Most Hydrophilic Bile Acid, in the Heart. Biomolecules.

[68] Gorelik J., Shevchuk A.I., Diakonov I., de Swiet M., Lab M., Korchev Y., Williamson C. (2003). Dexamethasone and Ursodeoxycholic Acid Protect against the Arrhythmogenic Effect of Taurocholate in an in Vitro Study of Rat Cardiomyocytes. BJOG.

[69] Abdul Kadir S.H.S., Ali N.N., Mioulane M., Brito-Martins M., Abu-Hayyeh S., Foldes G., Moshkov A.V., Williamson C., Harding S.E., Gorelik J. (2009). Embryonic Stem Cell-Derived Cardiomyocytes as a Model to Study Fetal Arrhythmia Related to Maternal Disease. J. Cell Mol. Med..

[70] Mohamed A.S., Hanafi N.I., Sheikh Abdul Kadir S.H., Md Noor J., Abdul Hamid Hasani N., Ab Rahim S., Siran R. (2017). Ursodeoxycholic Acid Protects Cardiomyocytes against Cobalt Chloride Induced Hypoxia by Regulating Transcriptional Mediator of Cells Stress Hypoxia Inducible Factor 1α and P53 Protein. Cell Biochem. Funct..

